# Model-based analysis of the incidence trends and transmission dynamics of COVID-19 associated with the Omicron variant in representative cities in China

**DOI:** 10.1186/s12889-023-17327-7

**Published:** 2023-12-02

**Authors:** Yifei Ma, Shujun Xu, Yuxin Luo, Jiantao Li, Lijian Lei, Lu He, Tong Wang, Hongmei Yu, Jun Xie

**Affiliations:** 1 School of Public Health, Shanxi Medical University, Taiyuan, 030001 China; 2 School of Management, Shanxi Medical University, Taiyuan, 030001 China; 3 Shanxi Provincial Key Laboratory of Major Diseases Risk Assessment, Taiyuan, 030001 China; 4 Center of Reverse Microbial Etiology, Shanxi Medical University, Taiyuan, 030001 China

**Keywords:** COVID-19, Omicron, SEAIQRD model, ARIMA model, LSTM model, Predictive performance, Transmission dynamics, Interventions

## Abstract

**Background:**

In 2022, Omicron outbreaks occurred at multiple sites in China. It is of great importance to track the incidence trends and transmission dynamics of coronavirus disease 2019 (COVID-19) to guide further interventions.

**Methods:**

Given the population size, economic level and transport level similarities, two groups of outbreaks (Shanghai *vs.* Chengdu and Sanya *vs.* Beihai) were selected for analysis. We developed the SEAIQRD, ARIMA, and LSTM models to seek optimal modeling techniques for waves associated with the Omicron variant regarding data predictive performance and mechanism transmission dynamics, respectively. In addition, we quantitatively modeled the impacts of different combinations of more stringent interventions on the course of the epidemic through scenario analyses.

**Results:**

The best-performing LSTM model showed better prediction accuracy than the best-performing SEAIQRD and ARIMA models in most cases studied. The SEAIQRD model had an absolute advantage in exploring the transmission dynamics of the outbreaks. Regardless of the time to inflection point or the time to *R*_t_ curve below 1.0, Shanghai was later than Chengdu (day 46 *vs.* day 12/day 54 *vs.* day 14), and Sanya was later than Beihai (day 16 *vs.* day 12/day 20 *vs.* day 16). Regardless of the number of peak cases or the cumulative number of infections, Shanghai was higher than Chengdu (34,350 *vs.* 188/623,870 *vs.* 2,181), and Sanya was higher than Beihai (1,105 *vs.* 203/16,289 *vs.* 3,184). Scenario analyses suggested that upgrading control level in advance, while increasing the index decline rate and quarantine rate, were of great significance for shortening the time to peak and *R*_t_ below 1.0, as well as reducing the number of peak cases and final affected population.

**Conclusions:**

The LSTM model has great potential for predicting the prevalence of Omicron outbreaks, whereas the SEAIQRD model is highly effective in revealing their internal transmission mechanisms. We recommended the use of joint interventions to contain the spread of the virus.

**Supplementary Information:**

The online version contains supplementary material available at 10.1186/s12889-023-17327-7.

## Background

As the most widespread epidemic in nearly a century and still raging worldwide, coronavirus disease 2019 (COVID-19) has infected more than 600 million people and killed approximately six million to date. The threat of this sudden and severe infectious disease to the whole world is arousing growing vigilance and awareness among people. The Omicron variant is by far the most dangerous strain, with greater transmissibility and immune escape than earlier variants that cause COVID-19 [1, 2], such as Alpha, Beta, Gamma and Delta. It has been shown that the effective reproduction number (*R*_t_) of the Omicron variant is 3.19 (95%CI: 2.82–3.61) times higher than that of Delta under the same epidemiological conditions [3], which is consistent with the findings of studies in South Africa and other countries [4]. The Omicron variant was first identified in South Africa on November 11, 2021, and by December 28, 2021, the total number of Omicron cases worldwide was 53,695, with 34,573 cases in the United Kingdom, 8,311 cases in the United States, 2,001 cases in Denmark, 1,643 cases in South Africa, 859 cases in Australia, 609 cases in Belgium, 586 cases in Canada, and 471 cases in Switzerland [5]. As of March 31, 2022, the Omicron variant has spread to 188 countries, and its rapid spread has caused great concern worldwide [6]. China’s first indigenous outbreak caused by Omicron was detected in Tianjin on January 11, 2022, and has now spread to Hong Kong, Taiwan, and 31 inland provinces. Influenza and COVID-19 are respiratory diseases with similar modes of transmission, but the *R*_t_ value of Omicron is much higher than that of influenza A and B (4.20 *vs.* 1.23) [7, 8]. A multicentre cohort study also suggested that Omicron infections are more common and associated with more severe outcomes than influenza and respiratory syncytial virus, especially in unvaccinated patients [9]. The above two points can reflect the high risk and great harm of Omicron variant. A recent study indicated that the Omicron variant will continue affecting the world, and the pandemic will not end until late 2023 [10]. Therefore, there is an urgent need to establish mathematical models to explore the incidence trends and transmission dynamics of Omicron. Our results not only lay the foundation for the scientific response and prevention of possible COVID-19 outbreaks in the future, but also provide an empirical reference for the global fight against pandemics triggered by the Omicron variant.

Here, we construct three models: the mechanism-driven theory-based infectious disease dynamics model, and the data-driven traditional statistical autoregressive integrated moving average (ARIMA) and deep learning long short-term memory (LSTM) models. These three models are widely recognized, well-established and extensively used forecasting models in the industry. The infectious disease dynamics model can be used to characterize the epidemiological patterns of diseases and evaluate the effectiveness of various prevention and control strategies through parametric sensitivity analysis, among which the susceptible-exposed-infected-recovered (SEIR) model occupies a central position [11, 12]. The ARIMA model can be applied to capture fluctuations in historical data, and the model itself is constructed with the help of endogenous variables, so it is well suited to address the interference of external factors. Furthermore, the model has the advantages of a simple structure, high applicability, and strong data interpretation ability and is widely used in the short-term prediction of infectious diseases [13, 14]. The LSTM model is a type of deep learning network that is able to “recall” patterns in past or future data without artificially adding temporal features and can explore the nonlinear correlation characteristics between time series data to a large extent [15, 16]. Given the unique strengths of the three models, we aimed to find the best-performing time series modeling techniques for COVID-19 in terms of 1)data predictive performance and 2)mechanism transmission dynamics, respectively.

In this study, the outbreaks in Shanghai, Chengdu, Sanya, and Beihai caused by Omicron subvariants BA.2, BA.2.76, BA.5.1.3, and BA.2.3 were selected for retrospective analysis among the several outbreaks that have occurred in China. Among them, Shanghai and Chengdu are the most economically developed and prosperous cities in eastern and central China, respectively, ranking in the top ten among all cities in terms of household resident population, gross regional product and airport passenger throughput. Sanya and Beihai are famous tourist cities in southern China, with total tourism revenue accounting for more than 80% and 40% of the gross regional product, respectively. The airport passenger throughput of the two cities ranks relatively high among all cities, but their household resident population and gross regional product rankings are very low. The demographic, geographic, economic, and transport profiles of the four cities are displayed in Additional file 1, Table S1, with data from the China City Statistical yearbook 2022, the National Civil Transport Airport Production Statistical Bulletin and the official websites of cities’ Bureau of Statistics. Based on accurate reviews of the outbreaks in the four representative cities, we compared the impacts of different prevention and control measures on the development of the epidemic in cities with comparable population sizes, economic levels and transport levels: Shanghai *vs.* Chengdu, and Sanya *vs.* Beihai, which contributes to drawing lessons from actual cases and optimizing control in a more targeted manner according to time and circumstances.

## Methods

### SEAIQRD model

**Fig. 1:**
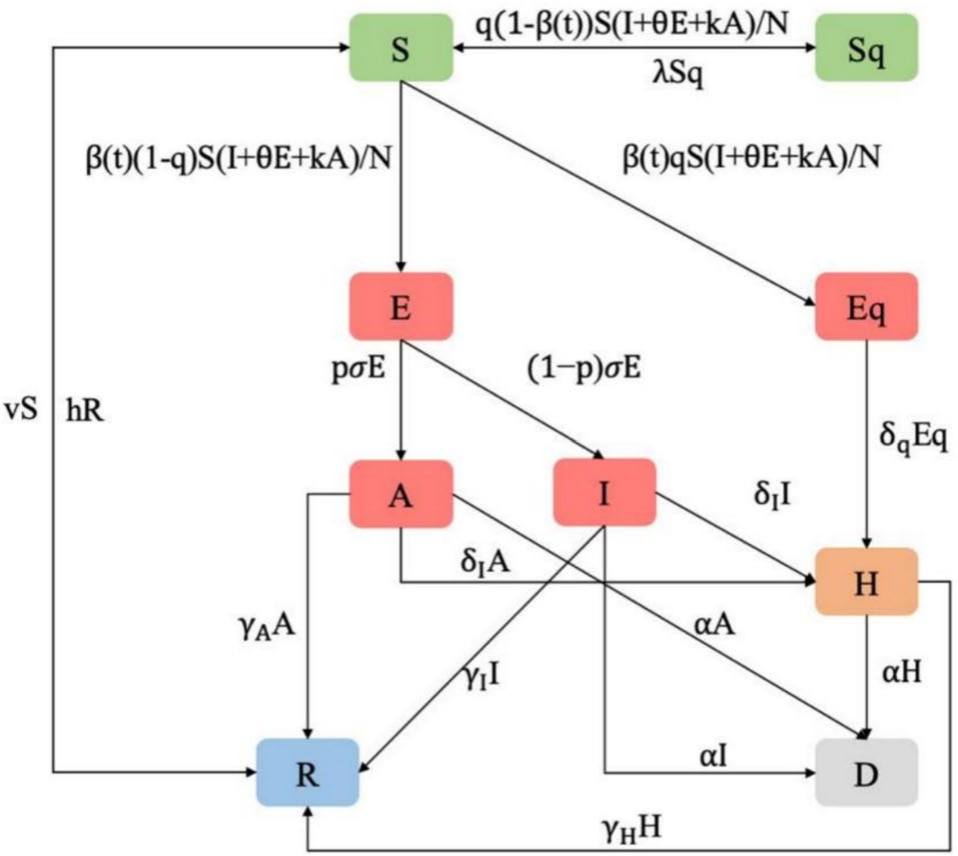
Illustration of the SEAIQRD model

We simulated the full transmission dynamics of COVID-19 in the four cities by extending the SEIR model to include asymptomatic (A), quarantined (Q) {quarantined susceptible (Sq), quarantined exposed (Eq), hospitalized (H)}, and dead (D), generating a model called SEAIQRD (Fig. 1). Let N(t) = S(t) + Sq(t) + E(t) + Eq(t) + A(t) + I(t) + H(t) + R(t) + D(t) be the total number of individuals in each city. The dynamics of the nine compartments at different times are described by the following system of nonlinear ordinary differential equations:

$$\begin{array}{l}\frac{dS}{dt}=\frac{-[\beta (t)+q(1-\beta (t))]S(I+\theta E+kA)}{N}+\lambda Sq-vS+hR\\ \frac{dSq}{dt}=\frac{q(1-\beta (t))S(I+\theta E+kA)}{N}-\lambda Sq\\ \begin{array}{c}\frac{dE}{dt}=\frac{\beta (t)(1-q)S(I+\theta E+kA)}{N}-\sigma E\\ \frac{dEq}{dt}=\frac{\beta (t)qS(I+\theta E+kA)}{N}-{\delta }_{q}Eq\\ \begin{array}{l}\frac{dA}{dt}=p\sigma E-{(\delta }_{I}+\alpha +{\gamma }_{A})A\\ \frac{dI}{dt}=(1-p)\sigma E-{(\delta }_{I}+\alpha +{\gamma }_{I})I\\ \begin{array}{l}\frac{dH}{dt}={\delta }_{I}(A+I)+{\delta }_{q}Eq-(\alpha +{\gamma }_{H})H\\ \frac{dR}{dt}={\gamma }_{A}A+{\gamma }_{I}I+{\gamma }_{H}H+vS-hR\\ \frac{dD}{dt}=\alpha (A+I+H)\end{array}\end{array}\end{array}\end{array}$$

#### Parameter simulation

The unknown parameters in the SEAIQRD model were estimated using the Markov Chain Monte Carlo-MH algorithm (MCMC-MH) [17], except for seven parameters, such as incubation rate, which were taken from actual epidemics or literature reports. The algorithm was iterated 90,000 times, and the Markov chain reached a stationary state after 75,000 burn-in periods. Diagnostic diagrams of the algorithm’s convergence are shown in Additional file 1, Fig. S1.

#### Segmented time-dependent transmission rate

To study time-varying trends and identify important changes during the COVID-19 epidemic, we performed a joinpoint regression (JPR) analysis [18]. For this study, JPR models were selected based on the modified Bayesian information criterion (MBIC). One of the advantages of this model is its ability to determine the number and location of inflection points, called “joinpoints”, over a period of time. In the SEAIQRD model, ${\beta }_{t}$ denotes the time-dependent transmission rate. We first applied JPR models to analyze the changing temporal patterns of infections in the four cities and then used the segmented function to characterize ${\beta }_{t}$ to better fit the actual epidemic development under the gradually strengthened prevention and control measures. ${\beta }_{t}$ is expressed as:

$$\beta (t)=\left\{\begin{array}{cc}{\beta }_{0}& 0\le t<{t}_{1}\\ {\beta }_{0}\times {e}^{-w(t-{t}_{1})}& {t}_{1}\le t<{t}_{2}\\ {\beta }_{0}\times {e}^{-w({t}_{2}-{t}_{1}-1)}\times {e}^{-r(t-{t}_{2})}& t\ge {t}_{2}\end{array}\right.$$

#### Basic reproduction number and effective reproduction number

The basic reproduction number (*R*_0_) is an important threshold parameter for measuring the transmission capacity and development trends of infectious diseases. In this study, *R*_0_ was expressed as the spectral radius of the next-generation matrix (NGM) by deriving the local stability of the disease-free equilibrium point [19]. According to the NGM combined with the SEAIQRD model and the time-dependent transmission rate, the *R*_t_ can be calculated. In the SEAIQRD model, three compartments are at risk of contagion, namely E, A, and I, so both F and V are third-order matrices:

$$\begin{array}{l}F=\left[\begin{array}{c}\frac{\beta (t)(1-q)S(I+\theta E+kA)}{N}\\ \begin{array}{c}0\\ 0\end{array}\end{array}\right]\\ \begin{array}{l}V=\left[\begin{array}{c}\sigma E\\ -p\sigma E+({\delta }_{I}+\alpha +{\gamma }_{A})A\\ -(1-p)\sigma E+({\delta }_{I}+\alpha +{\gamma }_{I})I\end{array}\right]\\ F(t)=\left[\begin{array}{ccc}\frac{\beta (t)(1-q)S\theta }{N}& \frac{\beta (t)(1-q)Sk}{N}& \frac{\beta (t)(1-q)S}{N}\\ 0& 0& 0\\ 0& 0& 0\end{array}\right]\\ \begin{array}{l}V(t)=\left[\begin{array}{ccc}\sigma & 0& 0\\ -p\sigma & {\delta }_{I}+\alpha +{\gamma }_{A}& 0\\ -\left(1-p\right)\sigma & 0& {\delta }_{I}+\alpha +{\gamma }_{I}\end{array}\right]\\ {FV}^{-1}=\left[\begin{array}{ccc}\frac{\beta (t)(1-q)S\theta }{N\sigma }+\frac{\beta (t)(1-q)Spk}{N{(\delta }_{I}+\alpha +{\gamma }_{A})}+\frac{\beta (t)(1-q)S(1-p)}{{N(\delta }_{I}+\alpha +{\gamma }_{I})}& \frac{\beta (t)(1-q)Sk}{N({\delta }_{I}+\alpha +{\gamma }_{A})}& \frac{\beta (t)(1-q)S}{{N(\delta }_{I}+\alpha +{\gamma }_{I})}\\ 0& 0& 0\\ 0& 0& 0\end{array}\right]\end{array}\end{array}\end{array}$$

*R*_t_ can be expressed as:

$${R}_{t}=\rho ({FV}^{-1})=\beta (t)(1-q)S[\frac{\theta }{N\sigma }+\frac{pk}{N({\delta }_{I}+\alpha +{\gamma }_{A})}+\frac{(1-p)}{N({\delta }_{I}+\alpha +{\gamma }_{I})}]$$

### ARIMA model

The ARIMA(*p*,*d*,*q*) model is a well-known linear time series forecasting method proposed by Box and Jenkins in the early 1970s, where AR is the autoregressive, *p* is the number of autoregressive terms, MA is the moving average, *q* is the number of moving average terms, and *d* is the number of differences required to become a stationary sequence. The ARIMA(*p*,*d*,*q*) model can be written as:

$$\left\{\begin{array}{c}\Phi (B){\nabla }^{d}{x}_{t}=\Theta (B){\varepsilon }_{t}\\ E({\varepsilon }_{t})=0,Var({\varepsilon }_{t})={{\sigma }_{\varepsilon }}^{2},E({\varepsilon }_{t}{\varepsilon }_{s})=0,s\ne t\\ E({x}_{s}{\varepsilon }_{t})=0,{\forall }_{s}<t\end{array}\right.$$

where ${\varepsilon }_{t}$ is the white noise sequence with expectation zero, $\Phi (B)=1-{\Phi }_{1}B-\cdots -{\Phi }_{p}{B}^{p}$ and $\Theta (B)=1-{\Theta }_{1}B-\cdots -{\Theta }_{q}{B}^{q}$ are the autoregressive and moving average components of the model, respectively.

The steps for constructing the ARIMA model are as follows. The original sequence is pre-processed using Augmented Dickey-Fuller (ADF) and Ljung-Box tests, and if it is not stable, it needs to be transformed into a stable non-white noise sequence by logarithmic transformation, differencing, etc. Subsequently, autocorrelation function (ACF) and partial autocorrelation function (PACF) plots are plotted for model identification, and the optimal model is ascertained by the Akaike information criterion (AIC) minimization. Finally, the residuals of the model are examined using the Ljung-Box test. If the residuals are white noise, the model is well-fitted, and the information is completely extracted [20].

### LSTM model

**Fig. 2:**
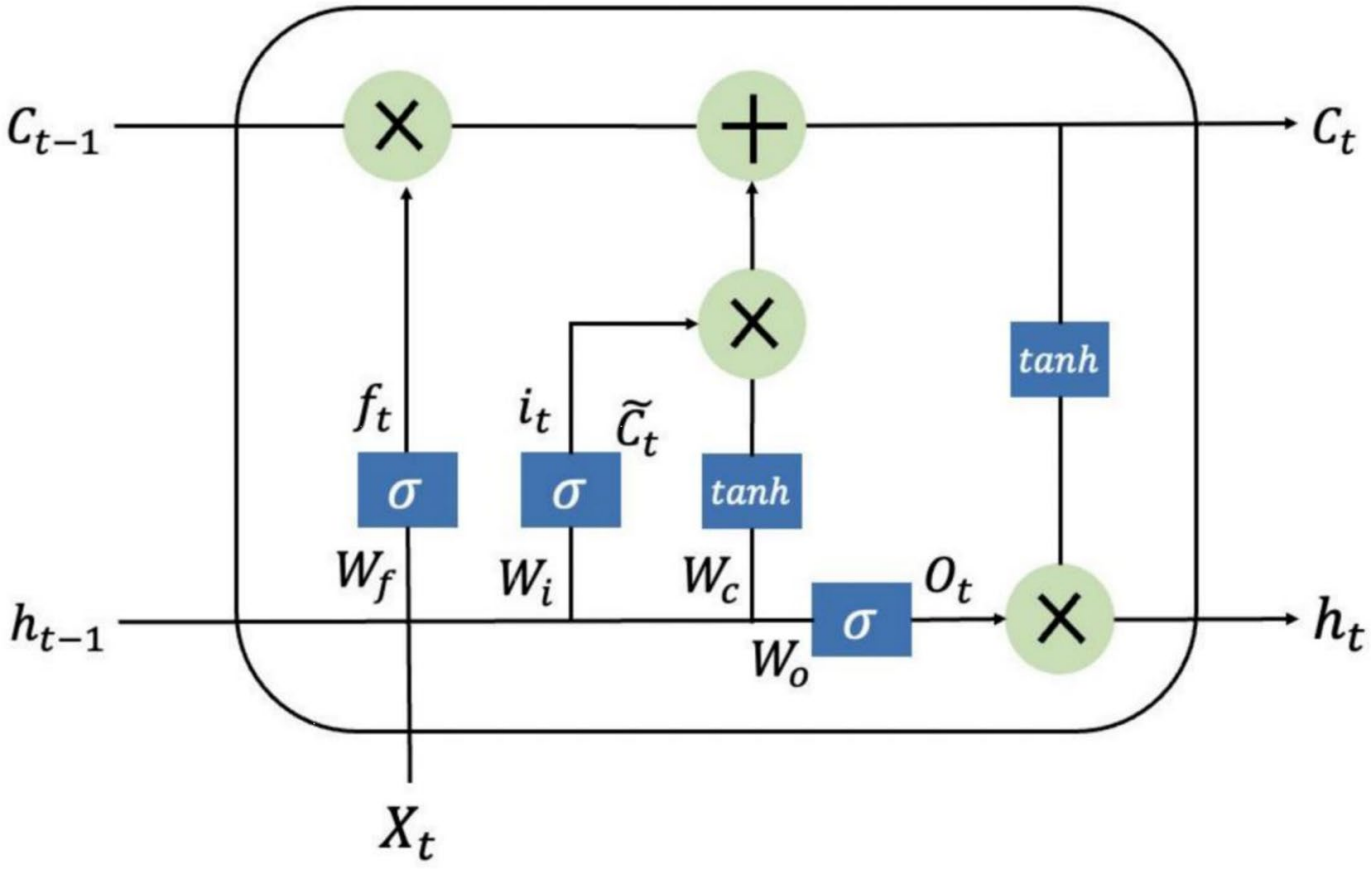
The inner structure of the LSTM model

The LSTM model has been used extensively to solve time series problems with long-dependent characteristics [21]. It is a special type of recurrent neural network (RNN) that avoids the situation of gradient disappearance or gradient explosion with the increase in network layers in traditional RNN [22]. The three gates (input, output, and forget gates) and cell state are the core concepts of LSTM. These gates can learn and decide what information can be added and stored or be forgot and removed during training. The cell state is responsible for storing and transferring long-term information down the sequence chain, which can be regarded as the “memory” of the neural network. The inner structure of the LSTM model is shown in Fig. 2, and the following estimated equations are used to define it:

$$\begin{array}{c}{f}_{t}=\sigma \left({W}_{f}\cdot \left[{h}_{t-1},{X}_{t}\right]+{b}_{f}\right)\\ {i}_{t}=\sigma \left({W}_{i}\cdot \left[{h}_{t-1},{X}_{t}\right]+{b}_{i}\right)\\ \begin{array}{c}\widetilde{{C}_{t}}=tanh\left({W}_{c}\cdot \left[{h}_{t-1},{X}_{t}\right]+{b}_{c}\right)\\ {C}_{t}={f}_{t}\cdot {C}_{t-1}+{i}_{t}\cdot \widetilde{{C}_{t}}\\ \begin{array}{c}{O}_{t}=\sigma \left({W}_{o}\cdot \left[{h}_{t-1},{X}_{t}\right]+{b}_{o}\right)\\ {h}_{t}={O}_{t}\cdot tanh\left({C}_{t}\right)\end{array}\end{array}\end{array}$$

where ${f}_{t},{i}_{t},{O}_{t}$ represent the forget gate, input gate, and output gate, respectively; $\widetilde{{C}_{t}}$ refers to the candidate memory cell states at time $t$; ${C}_{t}$ stands for the cell states at time $t$; ${h}_{t}$ is the hidden state at time $t$; $W$ is the weight matrix connecting the input signals; $b$ is the bias vector; $\sigma$ is the sigmoid activation function.

TensorFlow and Keras frameworks were used to build the LSTM model. First, before training the model, we applied maximum and minimum normalization method to restrict the data values between zero and one. Second, in the respective datasets of the four cities, the last seven days were split as the test set in prediction, while the rest were split into the training set. Third, the Particle Swarm Optimization (PSO) algorithm was used to find the preferred model, and the loss function was set to the mean squared error (MSE). Finally, the best set of hyperparameters was selected to produce out-of-sample predictions, and then the predicted values were normalized inverse.

### Measuring for accuracy

We limited the data analysis to March 1, 2022, to April 30, 2022, in Shanghai; August 22, 2022, to September 11, 2022, in Chengdu; August 1, 2022, to September 3, 2022, in Sanya; and July 12, 2022, to August 4, 2022, in Beihai, to construct prediction models and used the following seven days corresponding to each city for testing. Five error metrics, MSE, mean absolute error (MAE), root mean squared error (RMSE), mean absolute percentage error (MAPE), and root mean squared percentage error (RMSPE), were used to assess the predictive and simulative accuracy of the three models.

$$\begin{array}{l}MSE=\frac{1}{n}\sum_{i=1}^{n}{({y}_{i}-{\widehat{y}}_{i})}^{2}\\ MAE=\frac{1}{n}\sum_{i=1}^{n}|{y}_{i}-{\widehat{y}}_{i}|\\ \begin{array}{l}RMSE=\sqrt{\frac{1}{n}\sum_{i=1}^{n}{({y}_{i}-{\widehat{y}}_{i})}^{2}}\\ MAPE=\frac{1}{n}\sum_{i=1}^{n}|\frac{{y}_{i}-{\widehat{y}}_{i}}{{y}_{i}}|\times 100\%\\ RMSPE=\sqrt{\frac{1}{n}\sum_{i=1}^{n}{(\frac{{y}_{i}-{\widehat{y}}_{i}}{{y}_{i}})}^{2}}\times 100\%\end{array}\end{array}$$

where ${y}_{i}$ and ${\widehat{y}}_{i}$ represent the actual and predicted values, respectively, $n$ is the number of simulations and predictions in the models used.

## Results

### The best-performing SEAIQRD model

**Fig. 3:**
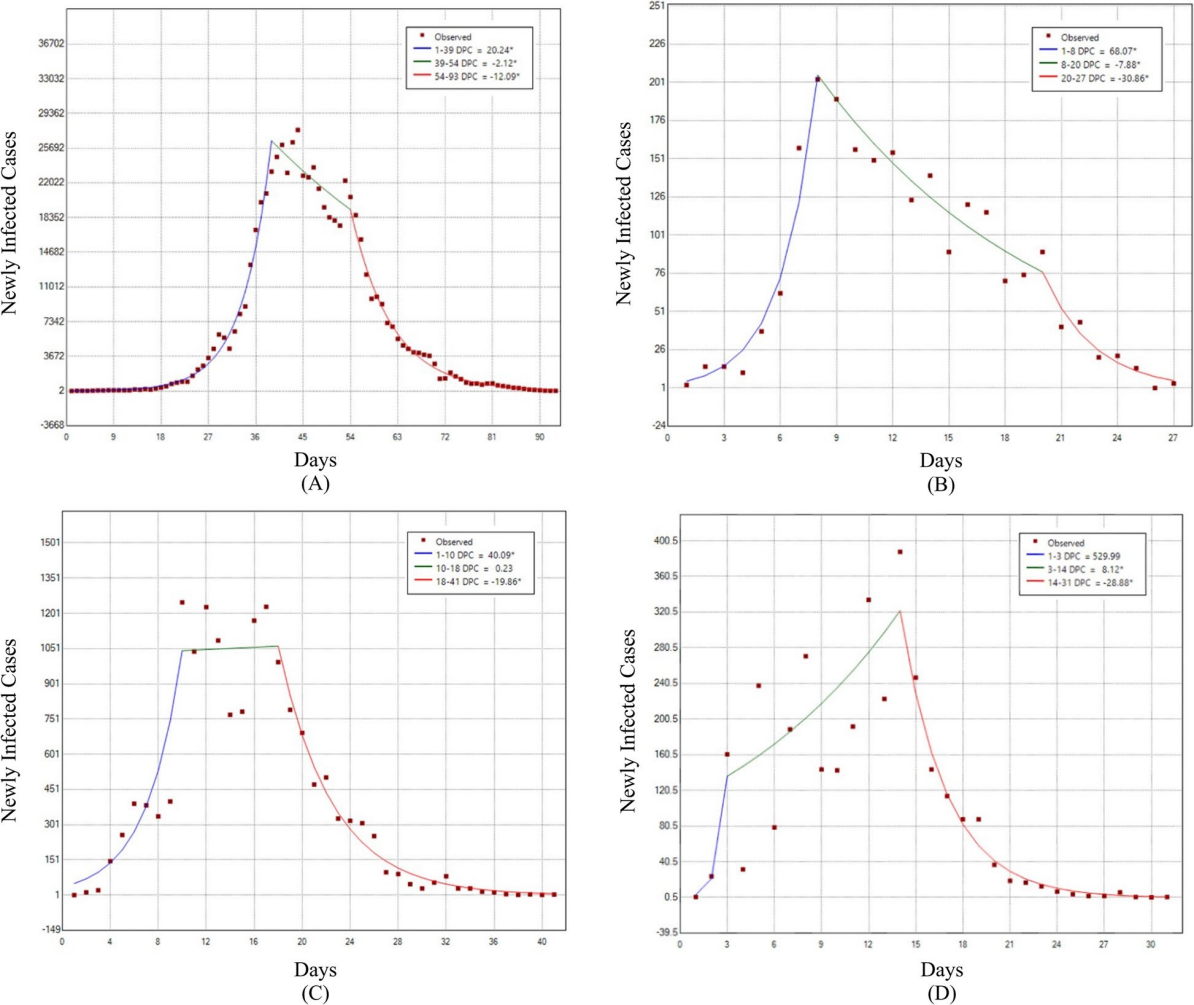
Joinpoint regression analysis of trends for COVID-19. **A** Shanghai, **B** Chengdu, **C** Sanya, **D** Beihai

The two joinpoints (Fig. 3) were observed on days 39/54 (Shanghai), 8/20 (Chengdu), 10/18 (Sanya), and 3/14 (Beihai), indicating three epidemic waves in these cities.

Table 1 shows the posterior means and 95% Bayesian credible intervals for the ten parameters. In this study, we considered the possibility of reinfection in recovered individuals due to decreased antibody titers, using $h$ to portray this process (R → S → A/I).

**Table 1:** Parameters settings for the four cities in the main analysis

Parameters	Shanghai value	Chengdu value	Sanya value	Beihai value	Source
${\beta }_{0}$	0.834 (0.822–0.845)	0.937 (0.930–0.945)	0.923 (0.912–0.938)	0.831 (0.819–0.839)	MCMC
$w$	0.098 (0.090–0.108)	0.307 (0.294–0.328)	0.167 (0.146–0.179)	0.092 (0.079–0.104)	MCMC
$r$	0.120 (0.111–0.131)	0.422 (0.411–0.434)	0.230 (0.211–0.251)	0.565 (0.553–0.579)	MCMC
${\delta }_{I}$	0.827 (0.809–0.839)	0.808 (0.789–0.825)	0.782 (0.767–0.795)	0.786 (0.777–0.798)	MCMC
${\delta }_{q}$	0.796 (0.780–0.813)	0.819 (0.808–0.832)	0.778 (0.761–0.792)	0.815 (0.805–0.821)	MCMC
${\gamma }_{A}$	0.079 (0.061–0.097)	0.095 (0.069–0.106)	0.076 (0.061–0.090)	0.110 (0.082–0.138)	MCMC
${\gamma }_{I}$	0.082 (0.061–0.100)	0.082 (0.063–0.105)	0.094 (0.077–0.105)	0.102 (0.087–0.122)	MCMC
${\gamma }_{H}$	0.071 (0.061–0.083)	0.124 (0.116–0.132)	0.071 (0.061–0.079)	0.128 (0.117–0.139)	MCMC
$\theta$	1.028 (1.016–1.039)	0.999 (0.990–1.009)	0.998 (0.984–1.009)	1.002 (0.987–1.026)	MCMC
$k$	0.985 (0.961–1.006)	1.011 (0.995–1.028)	1.024 (1.010–1.039)	1.007 (1.000–1.015)	MCMC
$\sigma$	1/4	1/4	1/4	1/4	Actual epidemic
$p$	0.941	0.379	0.639	0.899	Actual epidemic
$q$	0.150	0.070	0.130	0.120	Actual epidemic
$\alpha$	0.002	0.000	0.000	0.000	Actual epidemic
$\lambda$	1/14	1/14	1/14	1/14	Literature reports [23]
$v$	0.744	0.737	0.750	0.760	Actual epidemic
$h$	0.730	0.730	0.730	0.730	Literature reports [24]

${\beta }_{0}$: Initial transmission rate; $w$: Index decline rate (${t}_{1}\le t<{t}_{2}$); $r$: Index decline rate ($t\ge {t}_{2}$); ${\delta }_{I}$: Transition rate of infected cases to hospitalized cases; ${\delta }_{q}$: Transition rate of quarantined exposed cases to hospitalized cases; ${\gamma }_{A}$: Recovery rate of asymptomatic cases; ${\gamma }_{I}$: Recovery rate of confirmed cases; ${\gamma }_{H}$: Recovery rate of hospitalized cases; $\theta$: Ratio of transmission rate for exposed cases over confirmed cases; $k$: Ratio of transmission rate for asymptomatic cases over confirmed cases; $\sigma$: Incubation rate; $p$: Proportion of asymptomatic cases among infected cases; $q$: Quarantine rate; $\alpha$: Mortality rate of the virus; $\lambda$: Quarantine release rate; $v$: Immunity threshold (vaccination rate $\times$ vaccine protection rate); $h$: Decline rate of antibody titer.

### The best-performing ARIMA model

The ADF and Ljung-Box tests were performed on the original sequences (Table 2). We found that the unit roots existed in the four sequences (all *P*_*ADF*_ > 0.05), indicating non-stationary sequences. Subsequently, second-order, third-order, second-order, and second-order differences were carried out for Shanghai, Chengdu, Sanya, and Beihai, respectively, to obtain the stationary sequences. After differencing, the Ljung-Box test demonstrated that the four sequences satisfied the non-white noise requirement (all *P*_*Ljung-Box*_ < 0.05). Diagrams of the ACF and PACF of the original and difference sequences are shown in Additional file 1, Fig. S2 and S3. Based on the minimized AIC, ARIMA(0,2,1), ARIMA(1,3,0), ARIMA(1,2,0), and ARIMA(2,2,0) were selected as the optimal models for the four cities, with AIC values of 1463.59, 202.36, 449.31, and 272.39, respectively. The Q-Q plots show that most of the data points fell on straight lines, suggesting that the residuals were approximately normally distributed (Additional file 1, Fig. S4). Table 3 displays the parameter estimation and Ljung-Box test for the four ARIMA models. The parameters were found to be statistically significant (all *P* < 0.05), and the residuals were white noise sequences (all *P*_*Ljung-Box*_ > 0.05).

**Table 2:** ADF test and Ljung-Box test for sequences

City	Sequence	ADF test		Ljung-Box test	
		***χ***^***2***^	***P***	***χ***^***2***^	***P***
Shanghai	Before difference	-1.325	0.848	60.873	< 0.001
	After second-order difference	-4.614	< 0.01	8.904	0.003
Chengdu	Before difference	-1.313	0.834	14.243	< 0.001
	After third-order difference	-3.621	0.049	7.183	0.007
Sanya	Before difference	-1.986	0.579	26.898	< 0.001
	After second-order difference	-4.467	< 0.01	8.675	0.003
Beihai	Before difference	-1.329	0.828	7.181	0.007
	After second-order difference	-4.224	0.015	14.525	< 0.001

**Table 3:** Parameter estimation and Ljung-Box test for ARIMA models

City	Model	Parameter estimation					Ljung-Box test	
		**Variable**	***B***	***SE***	***t***	***P***	***χ***^***2***^	***P***
Shanghai	ARIMA(0,2,1)	MA(1)	0.784	0.085	9.206	< 0.001	0.086	0.769
Chengdu	ARIMA(1,3,0)	AR(1)	-0.584	0.206	-2.831	0.012	1.882	0.170
Sanya	ARIMA(1,2,0)	AR(1)	-0.483	0.159	-3.046	0.005	0.899	0.343
Beihai	ARIMA(2,2,0)	AR(1)	-1.122	0.194	-5.771	< 0.001	0.624	0.429
		AR(2)	-0.497	0.194	-2.569	0.019		

### The best-performing LSTM model

In this study, we selected simple LSTM models with one hidden layer and one fully connected layer because the four datasets were small. The time steps of the four LSTM networks were set to three, which meant that we used the data from the previous three days to forecast the newly infected cases on the next day. The PSO algorithm was applied to seek the best combinations of hyperparameters, including dropout rate, batch size, and hidden neurons. Four models were iterated over 500 epochs using an Adaptive Moment Estimation (Adam) optimizer. We confirmed that the preferred models with dropout rate = 0.05, batch size = 1, and hidden neurons = 15 (Shanghai) / 18 (Chengdu) / 16 (Sanya) / 12 (Beihai) had the lowest RMSE for the test set.

### Model comparison

#### Predictive performance

**Fig. 4:**
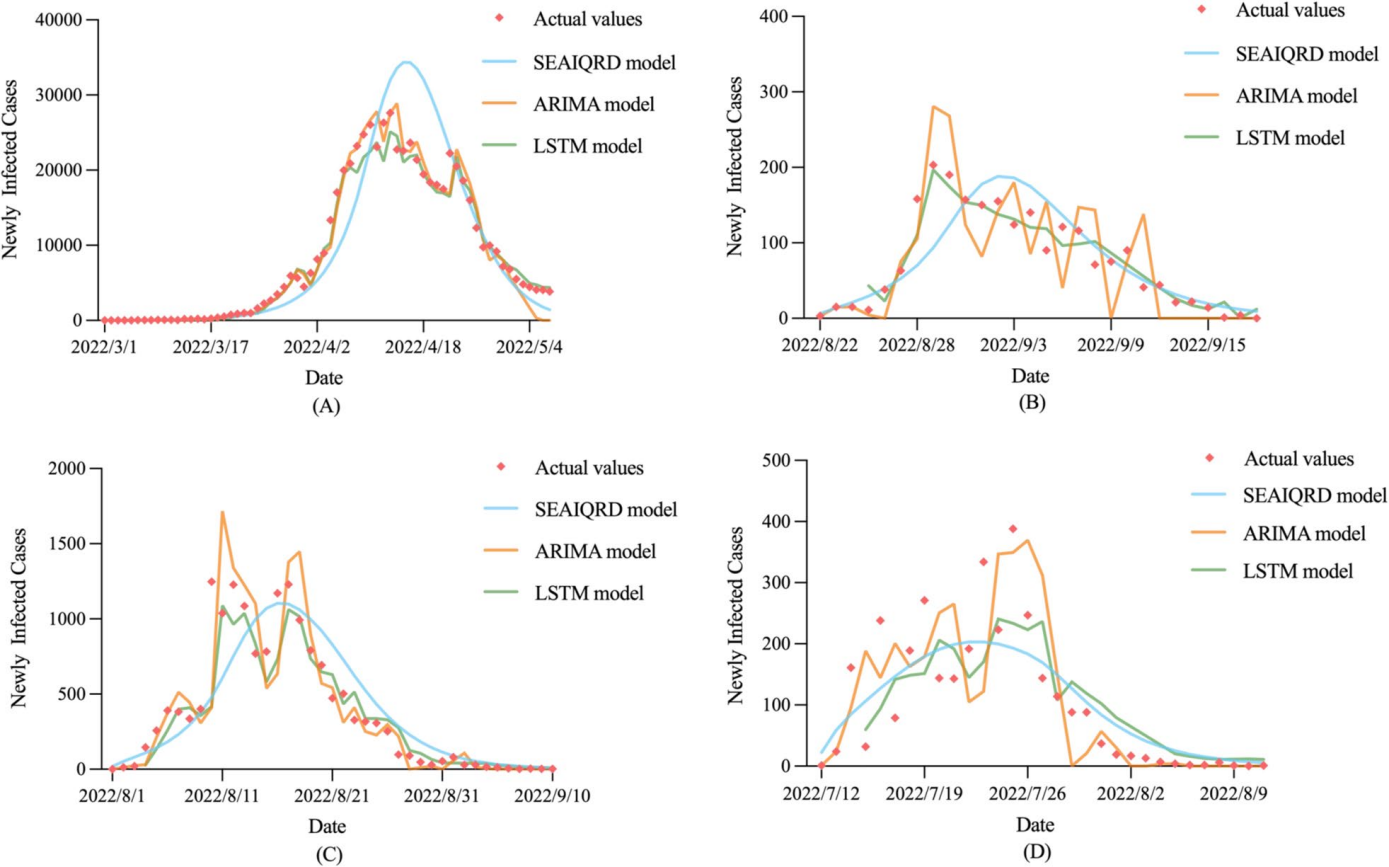
Newly infected cases of COVID-19 predicted by the three models in the four cities. **A** Shanghai, **B** Chengdu, **C** Sanya, **D** Beihai

The newly infected cases of COVID-19 predicted by the three models are shown in Fig. 4, where the predicted negative values were replaced by zero when plotted.

To objectively evaluate the predictive and simulative performance of the three models, we used five error metrics, MSE, MAE, RMSE, MAPE, and RMSPE, for comparison (Tables 4 and 5). In Shanghai and Sanya, the LSTM model showed better prediction accuracy than the SEAIQRD model, with the lowest MSE (532,966.69 *vs.* 3,175,990.71 and 198.65 *vs.* 319.86), MAE (661.93 *vs.* 1,729.47 and 12.84 *vs.* 16.53), RMSE (730.05 *vs.* 1,782.13 and 14.09 *vs.* 17.88), MAPE (0.14 *vs.* 0.39 and 2.99 *vs.* 3.85), as well as RMSPE (0.15 *vs.* 0.42 and 3.47 *vs.* 4.56). In Chengdu, the MAE was lower than that of SEAIQRD; however, the MSE and RMSE of LSTM was higher than that of SEAIQRD, contrasting to the case of Beihai, suggesting that the forecasting abilities of the two models were essentially the same in these two cities. However, the ARIMA model produced much worse forecasts for all four cities. Specific predicted values are listed in Additional file 1, Table S2. With respect to simulative performance, the values of the five error metrics of the LSTM model were lower than those of the other two models in most cases, indicating that the LSTM model could provide relatively reliable estimates of COVID-19 trends.

**Table 4:** Comparison of the predictive performance of the three models in the four cities

City	Model	Predictive performance				
		**MSE**	**MAE**	**RMSE**	**MAPE**	**RMSPE**
Shanghai	SEAIQRD	3,175,990.71	1,729.47	1,782.13	0.39	0.42
	ARIMA	13,231,814.70	3,108.64	3,637.56	0.74	0.90
	LSTM	532,966.69^a^	661.93^a^	730.05^a^	0.14^a^	0.15^a^
Chengdu	SEAIQRD	74.26^a^	7.72	8.55^a^	/	/
	ARIMA	473,119.62	561.51	687.84	/	/
	LSTM	91.80	7.23^a^	9.58	/	/
Sanya	SEAIQRD	319.86	16.53	17.88	3.85	4.56
	ARIMA	3,442.04	50.78	58.67	17.29	22.40
	LSTM	198.65^a^	12.84^a^	14.09^a^	2.99^a^	3.47^a^
Beihai	SEAIQRD	156.08	11.03^a^	12.49	/	/
	ARIMA	204.46	11.90	14.30	/	/
	LSTM	136.97^a^	11.23	11.70^a^	/	/

^a^Minimized MSE, MAE, RMSE, MAPE, and RMSPE of the three models

/ When zero is included in the actual values, the output of MAPE and RMSPE is /

**Table 5:** Comparison of the simulative performance of the three models in the four cities

City	Model	Simulative performance				
		**MSE**	**MAE**	**RMSE**	**MAPE**	**RMSPE**
Shanghai	SEAIQRD	22,614,818.98	2,840.19	4,755.50	0.31	0.42
	ARIMA	2,700,754.00	947.16^a^	1,643.40	0.15^a^	0.20^a^
	LSTM	2,646,314.86^a^	1,050.27	1,626.75^a^	0.99	2.23
Chengdu	SEAIQRD	1,798.64	29.16	42.41	0.38	0.55^a^
	ARIMA	2,995.71	44.76	54.73	0.52	0.74
	LSTM	481.49^a^	17.86^a^	21.94^a^	0.35^a^	0.78
Sanya	SEAIQRD	53,332.45	170.77	230.94	1.37	3.38
	ARIMA	60,430.88	152.58	245.83	0.44	0.66
	LSTM	42,689.32^a^	126.69^a^	206.61^a^	0.37^a^	0.57^a^
Beihai	SEAIQRD	4,786.40^a^	55.33^a^	69.18^a^	1.76	4.56
	ARIMA	9142.45	76.64	95.62	0.87	1.33
	LSTM	6736.12	67.63	82.07	0.64^a^	0.97^a^

^a^Minimized MSE, MAE, RMSE, MAPE, and RMSPE of the three models

#### Transmission dynamics

**Fig. 5:**
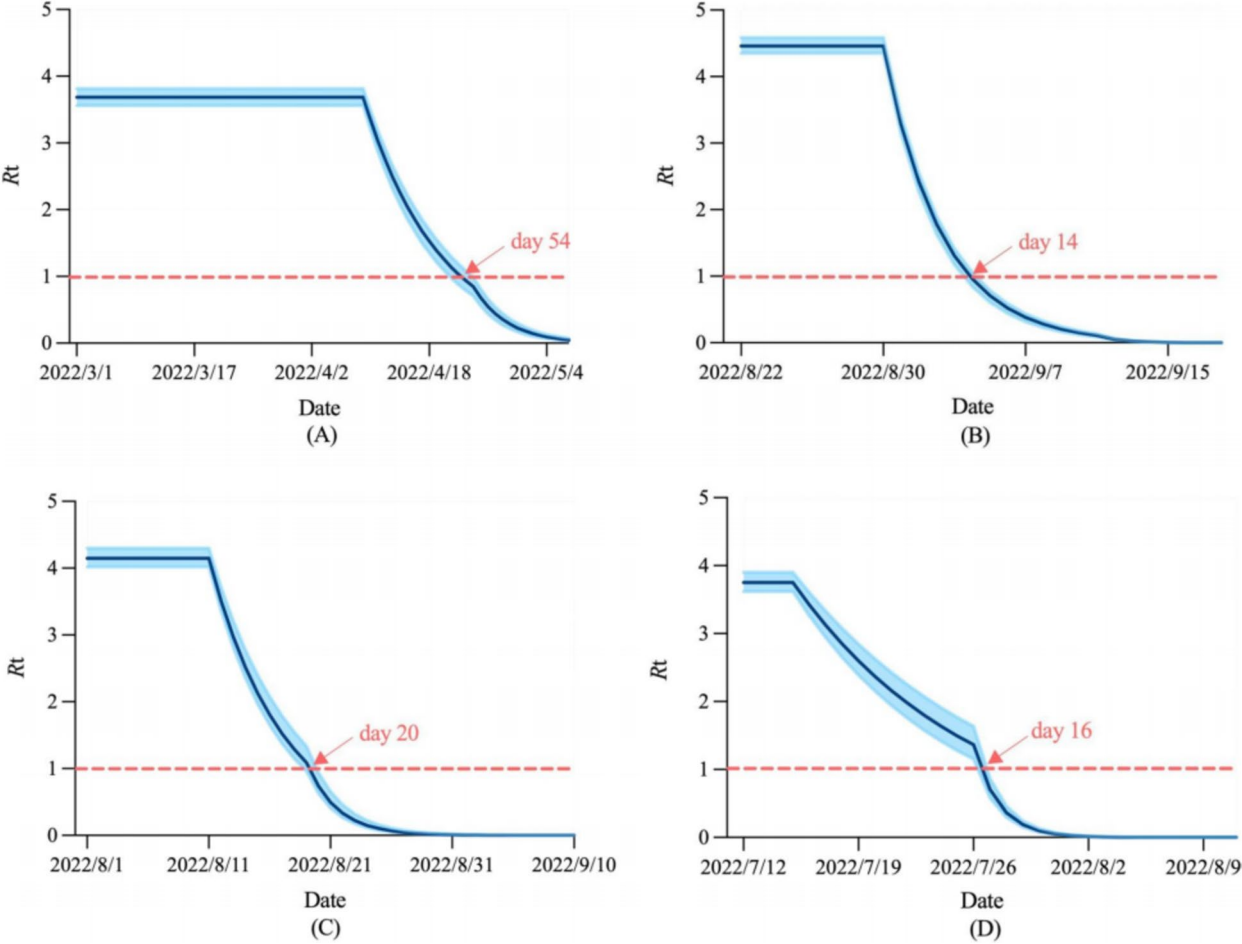
Trends of *R*_t_ in the four cities. **A** Shanghai, **B** Chengdu, **C** Sanya, **D** Beihai

The time taken to reach the peak, peak size, cumulative number of infections, and *R*_t_ are the indicators that have received the most attention since the outbreak [25]. Among the three models, the SEAIQRD had an absolute advantage in assessing the values of these indicators. The three major findings were as follows: 1) The outbreak in Shanghai peaked on day 46, 34 days later than that in Chengdu, with a peak size approximately 182.713 times that of Chengdu. Similarly, the outbreak in Sanya peaked on day 16, four days later than that in Beihai, with a peak size approximately 5.443 times that of Beihai. 2) In terms of the cumulative number of infections, Shanghai had 28,504.769% more than Chengdu, and Sanya had 411.589% more than Beihai. 3) In the absence of non-pharmaceutical interventions, the values of *R*_0_ for the four cities were 3.69 (95%CI: 3.56–3.82), 4.46 (95%CI: 4.35–4.59), 4.15 (95%CI: 4.02–4.30), and 3.75 (95%CI: 3.62–3.90), respectively. After the emergency response was carried out, *R*_t_ showed a gradual downward trend and dropped below 1.0 on April 23, 2022, September 4, 2022, August 20, 2022, and July 27, 2022. From the discovery of the first case to *R*_t_ below 1.0, Chengdu took only 14 days, 40 days shorter than Shanghai, and Beihai took only 16 days, four days shorter than Sanya (Table 6, Fig. 5).

**Table 6:** Values of epidemic indicators predicted by the SEAIQRD model in the four cities

City	Time taken to reach the peak	Peak size	Cumulative number of infections
Shanghai	April 15, 2022 (day 46)	34,350 (28,450–40,381)	623,870 (511,328–743,808)
Chengdu	September 2, 2022 (day 12)	188 (179–199)	2,181 (2,074–2,311)
Sanya	August 16, 2022 (day 16)	1,105 (1,036–1,183)	16,289 (15,191–17,596)
Beihai	July 23, 2022 (day 12)	203 (191–218)	3,184 (2,996–3,420)

### Scenario analysis

#### Time to upgrade the level of control

**Fig. 6:**
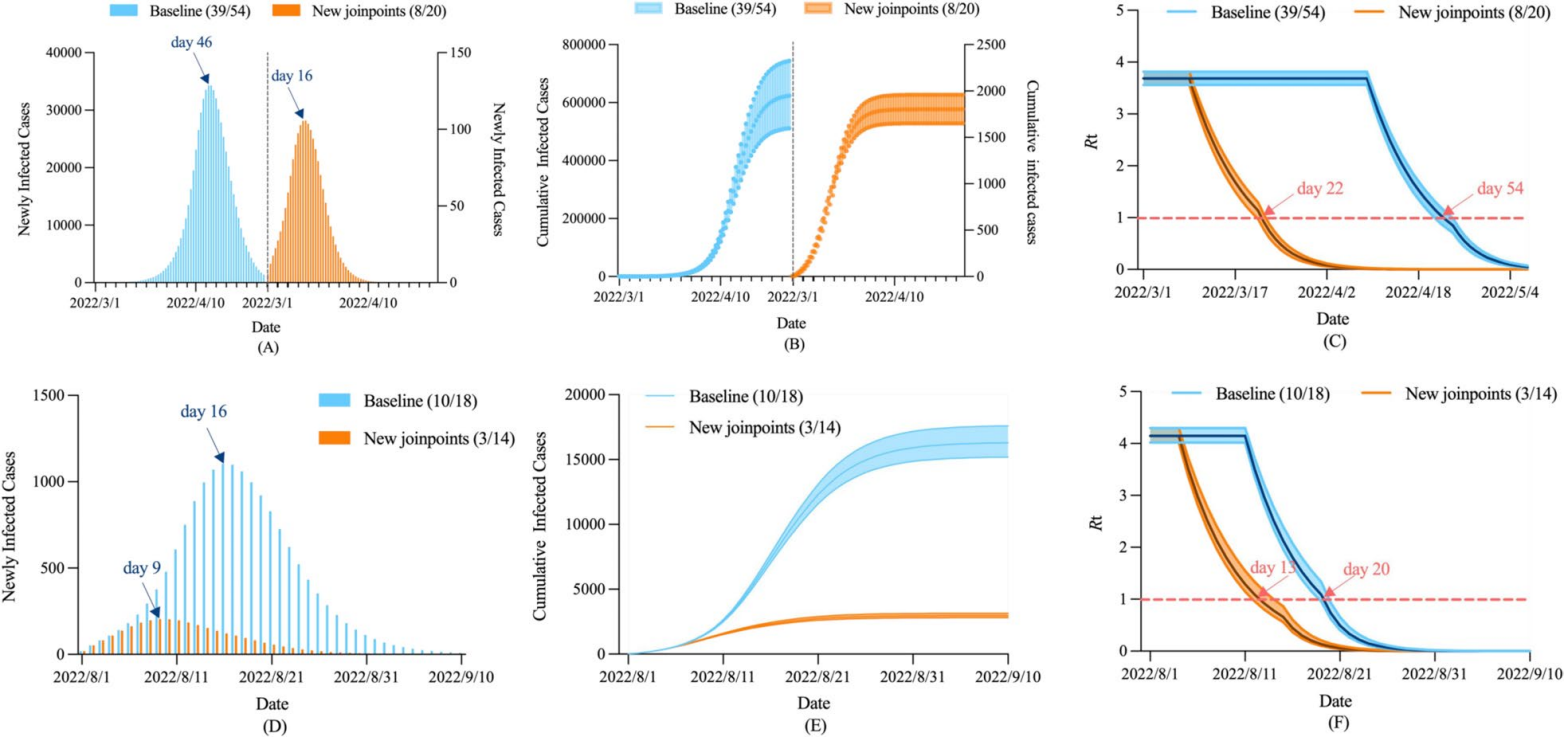
Scenario analysis of time to upgrade the level of control. **A** Newly infected cases in Shanghai, **B** Cumulative infected cases in Shanghai, **C** *R*_t_ in Shanghai, **D** Newly infected cases in Sanya, **E** Cumulative infected cases in Sanya, **F** *R*_t_ in Sanya

The above analysis shows that Shanghai was later than Chengdu and Sanya was later than Beihai, both in terms of the time to peak and the time to *R*_t_ below 1.0. Here, we attempted to simulate the number of infections in Shanghai and Sanya under the new joinpoints by replacing Shanghai with joinpoints in Chengdu (8/20) and Sanya with joinpoints in Beihai (3/14), respectively, while keeping other parameters unchanged. As shown in Fig. 6, when the time to upgrade the level of control was advanced, the peak daily new cases in Shanghai and Sanya were 0.308% and 18.580% of the baseline level, respectively, and peaked 30 and seven days earlier. The cumulative number of infected cases was only 1,803 (95%CI: 1,652–1,960) and 2,958 (95%CI: 2,806–3,146), a decrease of > 99% and 81%, respectively. The *R*_t_ curves for Shanghai and Sanya dropped below 1.0 32 and seven days ahead of schedule, respectively.

#### Stricter control measures

**Fig. 7:**
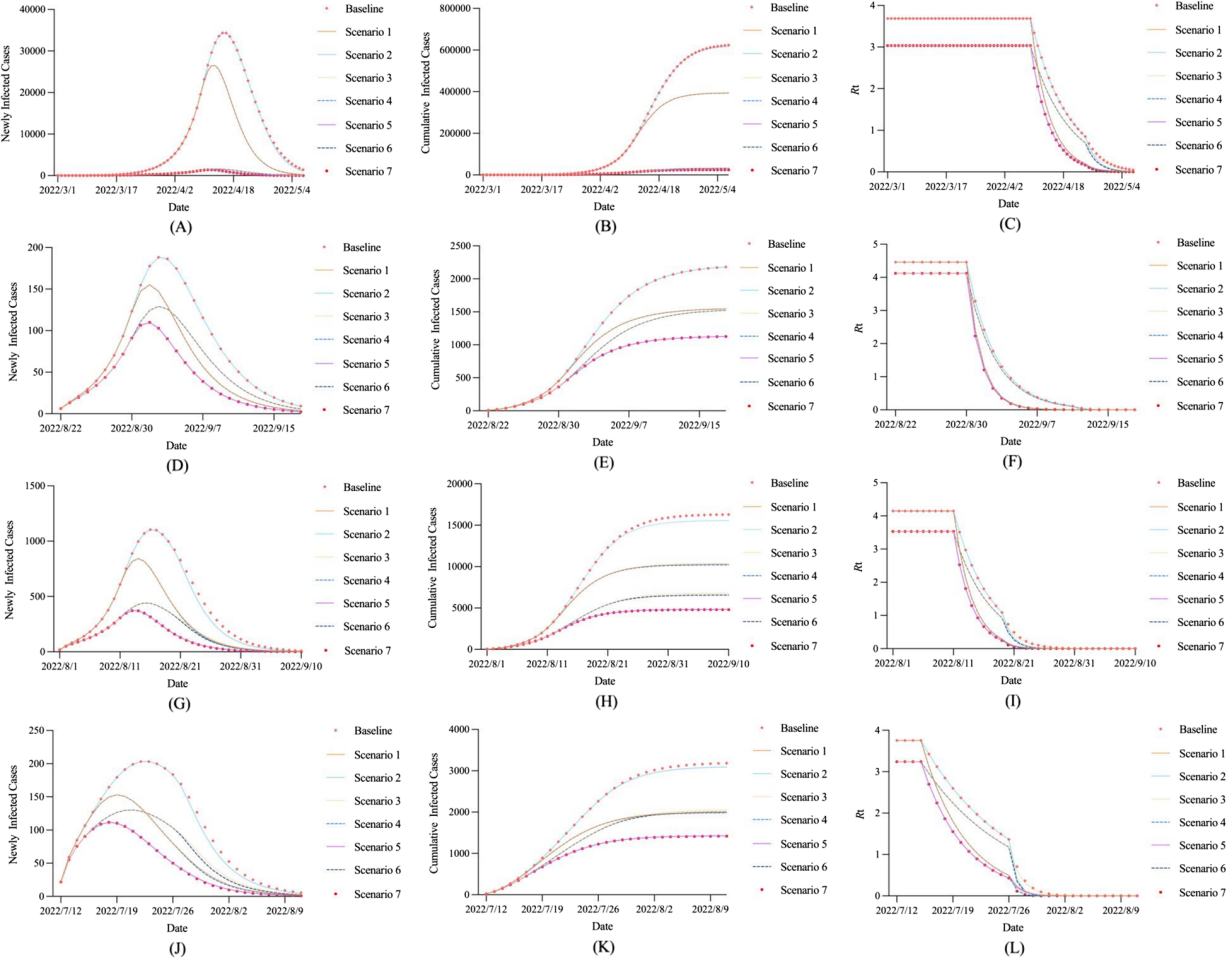
Scenario analysis of stricter control measure. **A** Newly infected cases in Shanghai, **B** Cumulative infected cases in Shanghai, **C** *R*_t_ in Shanghai, **D** Newly infected cases in Chengdu, **E** Cumulative infected cases in Chengdu, **F** *R*_t_ in Chengdu, **G** Newly infected cases in Sanya, **H** Cumulative infected cases in Sanya, **I** *R*_t_ in Sanya, **J** Newly infected cases in Beihai, **K** Cumulative infected cases in Beihai, **L** *R*_t_ in Beihai

In the SEAIQRD model, the values of the three parameters, $w$, $r$, and $q$, are closely related to the degree of implementation of the interventions to curb the spread of the virus. In this section, we performed seven scenario analyses to quantify the impacts of different parameters. Scenario 1: Double the value of parameter $w$ only. Scenario 2: Double the value of parameter $r$ only. Scenario 3: Double the value of parameter $q$ only. Scenario 4: Double the values of parameters $w$ and $r$. Scenario 5: Double the values of parameters $w$ and $q$. Scenario 6: Double the values of parameters $r$ and $q$. Scenario 7: Double the values of parameters $w$, $r$, and $q$ simultaneously. Figure 7 shows the trends of newly infected cases, cumulative infected cases, and *R*_t_ under different scenarios, and we found that Scenario 7 was always the optimal combination of prevention and control strategies in the four cities. Compared with the baseline, the beneficial results of implementing Scenario 7 were mainly reflected in two aspects. The first was the reduction in COVID-19 cases, with peak sizes in Shanghai, Chengdu, Sanya, and Beihai decreasing by 95.95%, 41.65%, 66.30%, and 45.09% from their respective baseline levels, and the final affected population decreasing by 96.08%, 48.40%, 70.49%, and 55.44%, respectively. The second was the advance in timing, with Shanghai, Chengdu, Sanya, and Beihai reaching inflection points three, one, three, and five days earlier than their respective baseline levels, and *R*_t_ below 1.0 eight, two, five, and five days earlier, respectively.

## Discussion

Since the Omicron variant became prevalent, several major cities in China have experienced outbreaks of COVID-19. Here, we retrospectively derived epidemic curves for four cities in search of optimal modeling techniques: first, in terms of data predictive performance, and second, in terms of mechanism transmission dynamics. In addition, we quantitatively simulated the effects of various combinations of intervention strategies to assist policymakers in coordinating responses to subsequent outbreaks.

We used three mature models to predict the incidence trends of COVID-19 in four representative cities, with the daily number of new infections as input. By comparing the predicted and simulated results, we found that the LSTM model outperformed the SEAIQRD and ARIMA models in the vast majority of the cases studied, and the performance of the ARIMA model was consistently worse. For a given time series, the ARIMA model is one of the optimal linear models driven by data and is suitable for predicting short-term epidemiological trends of infectious diseases [26]; however, its limitations are that it is difficult to capture the nonlinear characteristics of infectious disease data, and differential processing of raw data may lead to the under-utilization of information [27]. We also found that forecasting using the ARIMA model could easily result in negative values if the time series data showed a downward trend and there was no significant fluctuation after differencing; in this case, we had to use zero instead of negative values. Compared to the ARIMA model, the SEAIQRD model constructed in this study has a much higher predictive power. Firstly, within the framework of the classical SEIR model, we further added five compartments: quarantined susceptible, quarantined exposed, asymptomatic, hospitalized, and dead; secondly, we took into account the significance of non-conventional parameters such as the immune threshold and decline rate of antibody titer for virus transmission, and used the MCMC-MH algorithm to estimate the unknown parameters in the differential equations; finally, in order to characterize the effects of prevention and control measures, we used the JPR model to find “joinpoints” and set the transmission rate as a segmented function. At the early stage of the outbreak, due to the lack of in-depth understanding of COVID-19, scholars did not take into account the effects of vaccination, reduced antibody titer and the presence of a large number of asymptomatic infections on the transmission of the virus in their modeling, whereas the SEAIQRD model we constructed integrated multiple key factors, and thus the predictions were more in line with the actual situation [28, 29]. Nonetheless, in the mechanism-driven SEAIQRD model, the a priori information setting of the parameters is somewhat artificial and, therefore, inevitably subject to a certain deviation [30]. Therefore, a prerequisite for the use of infectious disease dynamics models is the need for good initial conditions and a good understanding of the disease under study [31]. Despite some drawbacks, dynamics models are useful for creating complex scenarios and performing analysis of epidemic tendency. In addition to speech recognition and video classification, LSTM deep learning technology has been applied to several research works on infectious diseases prediction. Instead of using predetermined rules regarding the disease transmission behavior, this technique defines rules centered on data. The advantages of this technique are the ability to capture the nonlinear dependencies of the time series data and the lack of requirements for the stability of the data itself, as well as the ability not only to preserve the general pattern of epidemic trends but also to identify occasional fluctuations [21]. This may explain why the predictive performances of the ARIMA and SEAIQRD models were less accurate than that of the LSTM model, and related studies have confirmed this result [26, 32–34]. Therefore, we recommended using the LSTM method when predicting the prevalence of COVID-19. While Rguibi et al. used statistical and artificial intelligence methods to predict short-term confirmed and fatal cases of COVID-19 in Morocco, respectively, our study, in addition to considering these two broad categories of methods, also developed predictions from the perspective of mechanism-driven infectious disease dynamics model, thus providing a more comprehensive and integrated comparison in terms of predictive performance [32].

In addition to considering the predictive performance of the three models, this study also explored the transmission dynamics of the COVID-19 epidemic in the four cities and estimated the values of relevant epidemic indicators, and then dissected the differences in prevention and control measures behind the number of infected people through comparative analysis. The mechanism-driven SEAIQRD model played an irreplaceable role in this process [35, 36]. We found that Shanghai was always later than Chengdu, and Sanya was always later than Beihai, regardless of the time to inflection point or the time to *R*_t_ curve below 1.0. Similarly, Shanghai was always higher than Chengdu, and Sanya was always higher than Beihai, regardless of the number of peak cases or the cumulative number of infections. It is worth noting that the *R*_0_ value was higher in Chengdu than in Shanghai and higher in Sanya than in Beihai, which was mainly related to the transmission capacity of the different mutant strains (BA.2.76 > BA.2, BA.5.1.3 > BA.2.3). In contrast to Chengdu and Beihai, Shanghai and Sanya did not respond promptly and effectively when the outbreak was initially detected, especially in Shanghai, where the government did not implement city-wide nucleic acid testing and widespread control until nearly a month after the virus had spread [30]. After modeling the Omicron outbreak in Shanghai in 2022, Yi et al. suggested that the course of the epidemic will depend on the efficiency of the implementation of public health interventions [37].

In the next section, we first simulated how the epidemic would have changed if Shanghai and Sanya were able to respond in advance: Shanghai ahead to coincide with Chengdu (8/20) and Sanya ahead to coincide with Beihai (3/14). The simulation results showed that Shanghai and Sanya would reach the inflection point as well as *R*_t_ below 1.0, approximately one month and one week earlier, respectively, while both peak cases and the final affected population were significantly reduced compared to the baseline. This suggests that the earlier timing of upgrading the control level has a significant effect on inhibiting the further spread of COVID-19. The results of one of our previous studies also showed that the earlier the control measures are implemented, the sooner the turning point of the epidemic will arrive [30]. Subsequently, we explored the impacts of different combinations of stricter control measures on the epidemic in the four cities through seven scenario analyses. Of these, Scenario 7, which simultaneously increased the index decline rates in the first and second stages ($w$, $r$) and the quarantine rate ($q$), was the optimal strategy. Furthermore, we found that increasing the index decline rate in stage 2 alone, compared to increasing the index decline rate in stage 1 or the quarantine rate, did not have much impact on the epidemic. Another study also suggested that the ultimate effect of controlling the outbreak through only one intervention was not significant [12]. Therefore, we recommended that joint interventions, such as the timely isolation of high-risk groups, early upgrading of control levels, and a significant reduction in transmission rates, be used to rapidly control the outbreak and ultimately reduce the total number of cases.

This study has several limitations. First, although the LSTM model established can be regarded as a practical tool for COVID-19 trend forecasting, in practice, this model should be updated in due course according to different conditions and time periods to ensure its high predictive performance. Second, although the aim of this study was to construct three widely recognized, well-established and extensively used models from the theoretical, statistical and deep learning domains to explore the incidence trends and transmission dynamics of Omicron, some new deep learning models that have emerged in recent years have also demonstrated good performance in predicting COVID-19 trends, such as Temporal Convolutional Network (TCN). Given the shortcoming that the sample size of this study is too small, in the future, we will focus on collecting more epidemic data to expand the sample size, and using the TCN model in multiple scenarios to make it applicable to more complex and variable infection situations. Third, this paper currently focuses only on analyzing the development of outbreaks in four representative cities triggered by the Omicron variant in 2022. In the future, we need to consider including cities with different characteristics in the scope of the study and conducting further research at the national or even global level to provide a more comprehensive perspective. Finally, only non-pharmaceutical interventions, such as upgraded control, tracking and isolating were considered, whereas the impact of increased awareness of self-protection on the epidemic has not been assessed.

## Conclusions

In this study, three mature models were established to track the incidence trends and transmission dynamics of COVID-19 in mainland China. Among them, the LSTM model can provide more accurate predictions, and the SEAIQRD model can reveal internal transmission mechanisms. Moreover, reducing the transmission rate of the virus and increasing the quarantine rate of high-risk groups, coupled with upgrading control levels as soon as possible, could quickly contain the spread of the outbreak. These findings not only help health departments prepare in advance for a possible outbreak of COVID-19 but also provide an important reference for optimizing non-pharmaceutical intervention programs.

### Supplementary Information

**Additional file 1:** **Fig. S1.** Diagnostic diagrams of the convergence of the algorithm. (A) Shanghai, (B) Chengdu, (C) Sanya, (D) Beihai. **Fig. S2.** Diagrams of the ACF and PACF of the original sequences. (A) Shanghai ACF, (B) Shanghai PACF, (C) Chengdu ACF, (D) Chengdu PACF, (E) Sanya ACF, (F) Sanya PACF, (G) Beihai ACF, (H) Beihai PACF. **Fig. S3.** Diagrams of the ACF and PACF of the difference sequences. (A) Shanghai ACF, (B) Shanghai PACF, (C) Chengdu ACF, (D) Chengdu PACF, (E) Sanya ACF, (F) Sanya PACF, (G) Beihai ACF, (H) Beihai PACF. **Fig. S4.** Q-Q plots of the residuals of the ARIMA models. (A) Shanghai, (B) Chengdu, (C) Sanya, (D) Beihai. **Table S1.** Demographic, geographic, economic, and transport profiles of the four cities. **Table S2.** Actual and predicted values of the three models in the four cities.

**Additional file 2.** 

## Data Availability

The datasets analyzed during the current study are available in the supplementary material.
