# Supplementary material for: Model-based analysis of the incidence trends and transmission dynamics of COVID-19 associated with the Omicron variant in representative cities in China
Source: BMC Public Health. 2023 Dec 2;23:2400. doi: 10.1186/s12889-023-17327-7 (PMC10693062; doi:10.1186/s12889-023-17327-7)
Supplement: Supplementary file 1 — Additional file 1: Fig. S1. Diagnostic diagrams of the convergence of the algorithm. (A) Shanghai, (B) Chengdu, (C) Sanya, (D) Beihai. Fig. S2. Diagrams of the ACF and PACF of the original sequences. (A) Shanghai ACF, (B) Shanghai PACF, (C) Chengdu ACF, (D) Chengdu PACF, (E) Sanya ACF, (F) Sanya PACF, (G) Beihai ACF, (H) Beihai PACF. Fig. S3. Diagrams of the ACF and PACF of the difference sequences. (A) Shanghai ACF, (B) Shanghai PACF, (C) Chengdu ACF, (D) Chengdu PACF, (E) Sanya ACF, (F) Sanya PACF, (G) Beihai ACF, (H) Beihai PACF. Fig. S4. Q-Q plots of the residuals of the ARIMA models. (A) Shanghai, (B) Chengdu, (C) Sanya, (D) Beihai. Table S1. Demographic, geographic, economic, and transport profiles of the four cities. Table S2. Actual and predicted values of the three models in the four cities. [file 12889_2023_17327_MOESM1_ESM.docx]

**
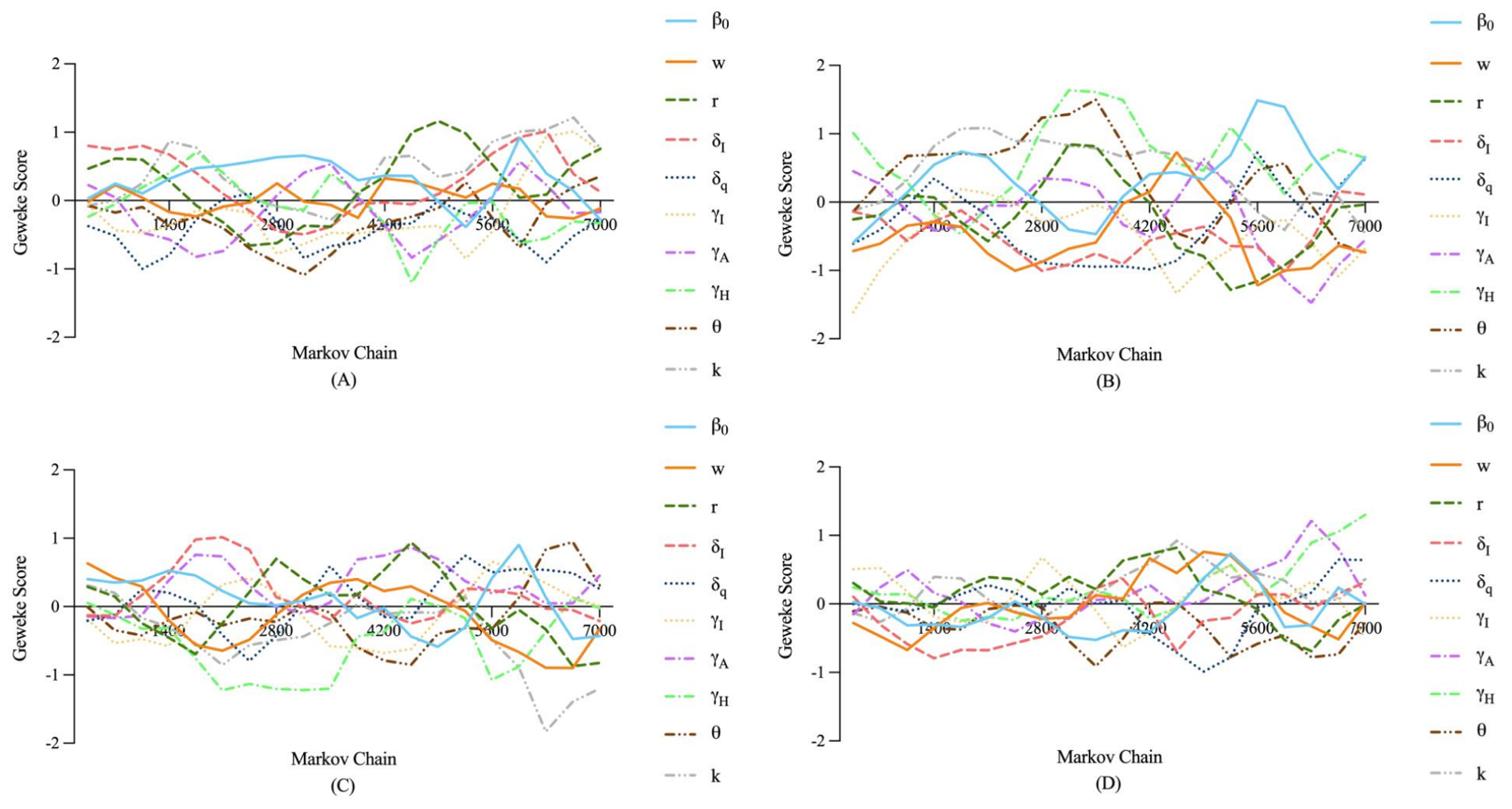
Fig. S1** Diagnostic diagrams of the convergence of the algorithm. (A) Shanghai, (B) Chengdu, (C) Sanya, (D) Beihai

**
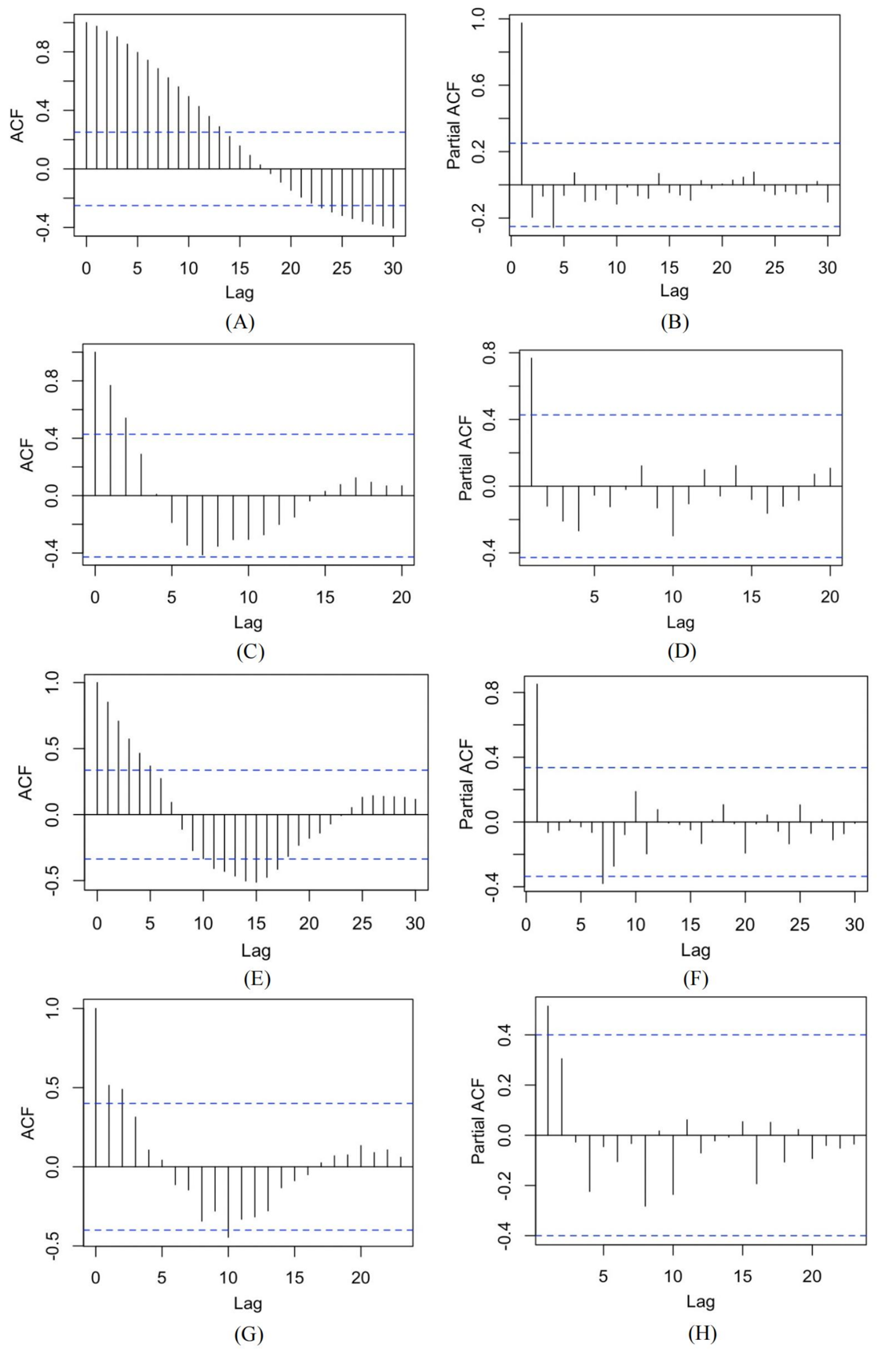
Fig. S2** Diagrams of the ACF and PACF of the original sequences. (A) Shanghai ACF, (B) Shanghai PACF, (C) Chengdu ACF, (D) Chengdu PACF, (E) Sanya ACF, (F) Sanya PACF, (G) Beihai ACF, (H) Beihai PACF

**
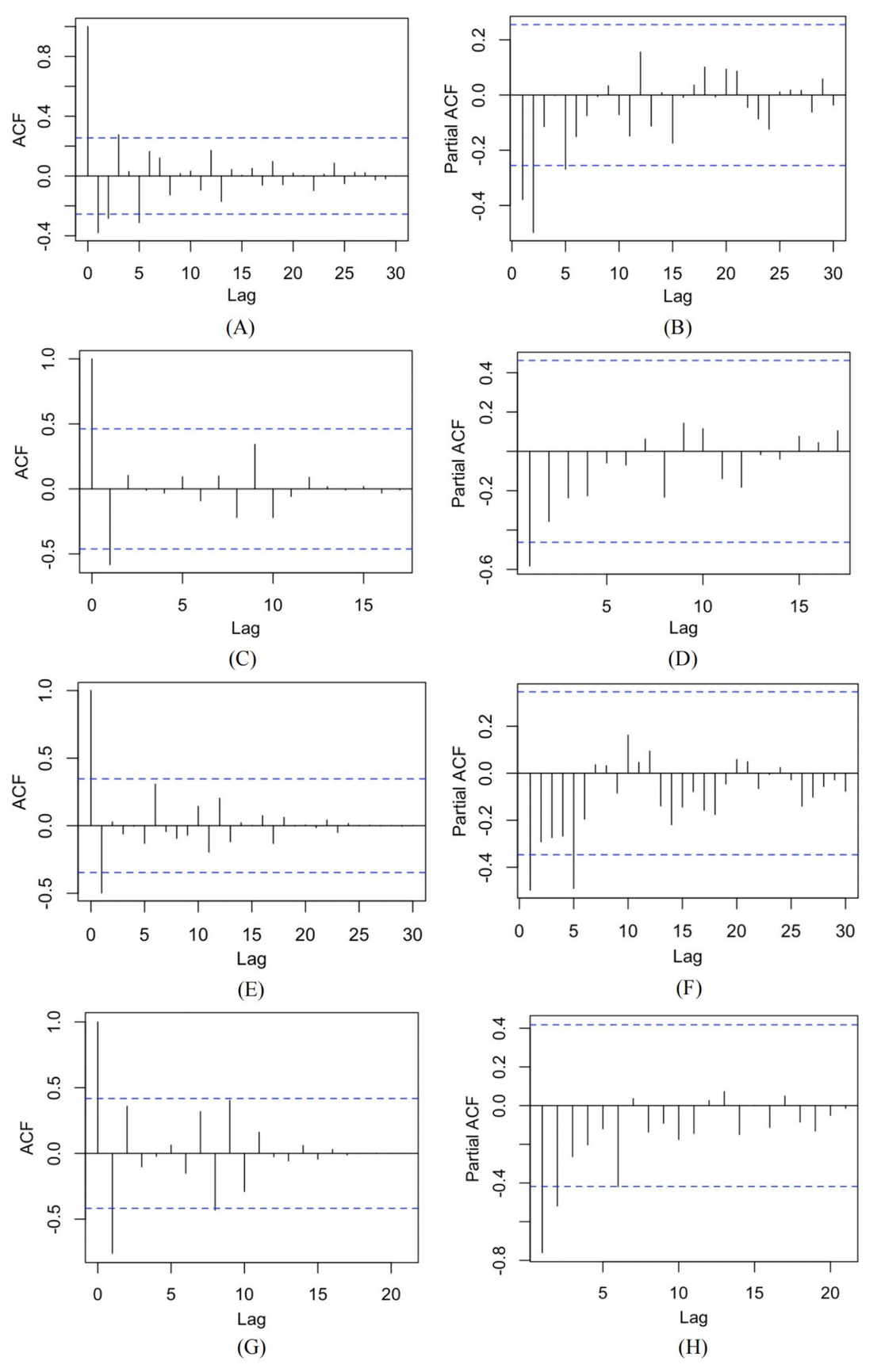
Fig. S3** Diagrams of the ACF and PACF of the difference sequences. (A) Shanghai ACF, (B) Shanghai PACF, (C) Chengdu ACF, (D) Chengdu PACF, (E) Sanya ACF, (F) Sanya PACF, (G) Beihai ACF, (H) Beihai PACF

**
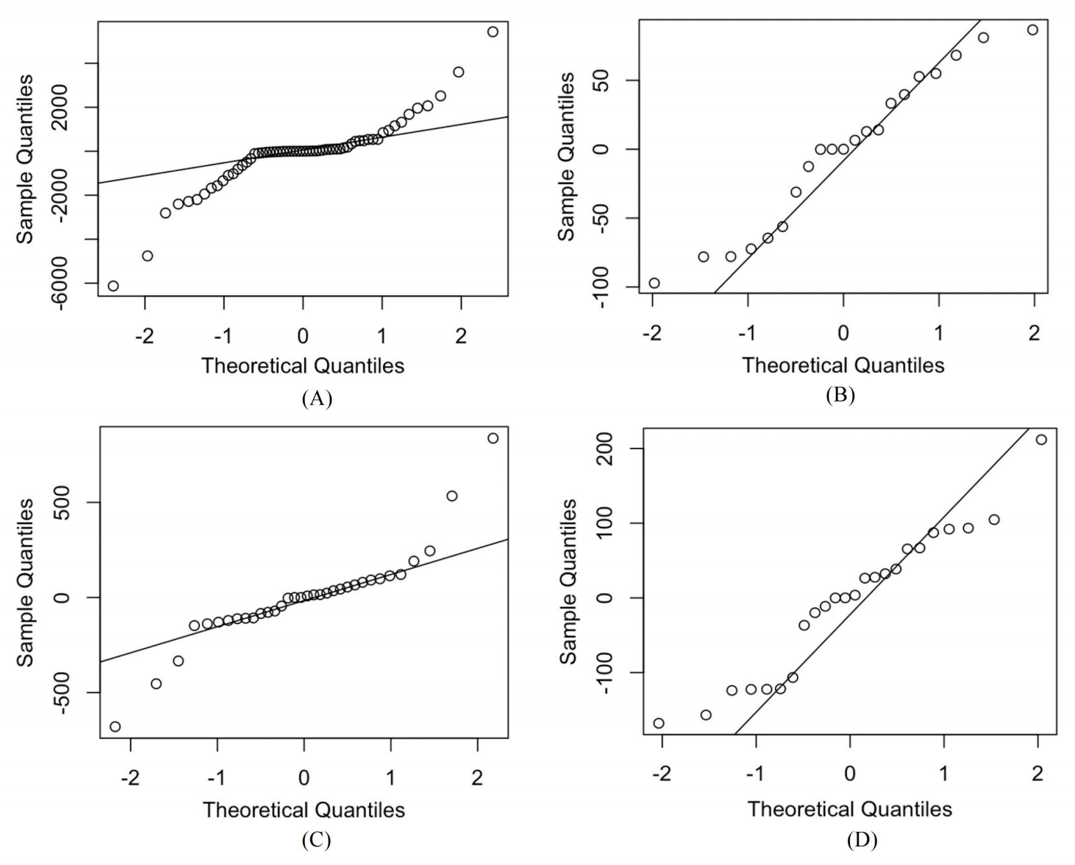
Fig. S4** Q-Q plots of the residuals of the ARIMA models. (A) Shanghai, (B) Chengdu, (C) Sanya, (D) Beihai

**Table S1** Demographic, geographic, economic, and transport profiles of the four cities

| **City** | **Household resident population**  **(10 000 persons) / ranking** | **Geographic location** | **Gross regional product**  **(billions) / ranking** | **Airport passenger throughput**  **(10 000 persons) / ranking** |
| --- | --- | --- | --- | --- |
| Shanghai | 1,493 / 3 | 30°40’-31°53’N, 120°52’-122°12’E | 43,215 / 1 | 6,541 / 1 |
| Chengdu | 1,556 / 2 | 30°05’-31°26’N, 102°54’-104°53’E | 19,917 / 7 | 4,447 / 3 |
| Sanya | 71 / 285 | 18°09’-18°37’N, 108°56’-109°48’E | 835 / 250 | 1,663 / 17 |
| Beihai | 183 /241 | 20°26’-21°55’N, 108°50’-109°47’E | 1,504 / 192 | 189 / 60 |

**Table S2** Actual and predicted values of the three models in the four cities

| **City** | **Date** | **Actual values** | **SEAIQRD**  **predicted values** | **ARIMA**  **predicted values** | **LSTM**  **predicted values** |
| --- | --- | --- | --- | --- | --- |
|  | 1 May, 2022 | 6,804 | 5,339.262 | 5,813.948 | 7,138.369 |
|  | 2 May, 2022 | 5,514 | 4,339.867 | 4,438.896 | 6,752.107 |
|  | 3 May, 2022 | 4,831 | 3,505.986 | 3,063.845 | 5,812.489 |
| Shanghai | 4 May, 2022 | 4,466 | 2,817.251 | 1,688.793 | 4,956.404 |
|  | 5 May, 2022 | 4,088 | 2,253.330 | 313.741 | 4,757.290 |
|  | 6 May, 2022 | 4,039 | 1,795.033 | -1,061.311 | 4,423.961 |
|  | 7 May, 2022 | 3,840 | 1,424.953 | -2,436.363 | 4,374.878 |
|  | 12 September, 2022 | 44 | 40.042 | -28.188 | 40.558 |
|  | 13 September, 2022 | 21 | 31.656 | -143.157 | 26.066 |
| Chengdu | 14 September, 2022 | 22 | 24.907 | -288.956 | 17.199 |
|  | 15 September, 2022 | 14 | 19.522 | -474.319 | 12.427 |
|  | 16 September, 2022 | 1 | 15.258 | -694.145 | 21.579 |
|  | 17 September, 2022 | 4 | 11.901 | -951.413 | 0.960 |
|  | 18 September, 2022 | 0 | 9.269 | -1,244.383 | 12.118 |
|  | 4 September, 2022 | 15 | 42.742 | 3.894 | 39.238 |
|  | 5 September, 2022 | 13 | 33.332 | -9.091 | 26.390 |
|  | 6 September, 2022 | 5 | 25.968 | -27.928 | 22.014 |
| Sanya | 7 September, 2022 | 2 | 20.216 | -43.939 | 14.533 |
|  | 8 September, 2022 | 4 | 15.728 | -61.315 | 10.375 |
|  | 9 September, 2022 | 2 | 12.230 | -78.032 | 11.362 |
|  | 10 September, 2022 | 3 | 9.507 | -95.067 | 9.996 |
|  | 5 August, 2022 | 4 | 25.056 | 4.238 | 20.381 |
|  | 6 August, 2022 | 2 | 19.492 | -1.160 | 16.134 |
|  | 7 August, 2022 | 2 | 15.150 | -5.213 | 12.773 |
| Beihai | 8 August, 2022 | 6 | 11.770 | -9.463 | 11.127 |
|  | 9 August, 2022 | 1 | 9.142 | -14.161 | 11.471 |
|  | 10 August, 2022 | 0 | 7.099 | -18.259 | 11.908 |
|  | 11 August, 2022 | 1 | 5.512 | -22.807 | 10.843 |
